# The Hare and the Hedgehog: Empirical evidence on the relationship between the individual *Pace of Life* and the speed-accuracy continuum

**DOI:** 10.1371/journal.pone.0256490

**Published:** 2021-08-20

**Authors:** Christin Hoffmann, Julia Amelie Hoppe, Niklas Ziemann

**Affiliations:** 1 Chair of General Business Administration, Especially Aspects of Organisation and Corporate Governance, Faculty of Business, Law and Social Sciences, Brandenburg University of Technology, Cottbus, Germany; 2 Chair of Organizational Behavior, Faculty of Business Administration and Economics, University of Paderborn, Paderborn, Germany; 3 Chair of Economics, especially Markets, Competition and Institutions, Faculty of Economics and Social Sciences, University of Potsdam, Potsdam, Germany; Julius-Maximilians-Universität Würzburg, GERMANY

## Abstract

Against the background of the speed-accuracy trade-off, we explored whether the *Pace of Life* can be used to identify heterogeneity in the strategy to place more weight on either fast or accurate accomplishments. The *Pace of Life* approaches an individual’s exposure to time and is an intensively studied concept in the evolutionary biology research. Albeit overall rarely, it is increasingly used to understand human behavior and may fulfill many criteria of a personal trait. In a controlled laboratory environment, we measured the participants’ *Pace of Life*, as well as their performance on a real-effort task. In the real-effort task, the participants had to encode words, whereby each word encoded correctly was associated with a monetary reward. We found that individuals with a faster *Pace of Life* accomplished *more* tasks in total. At the same time, they were *less* accurate and made more mistakes (in absolute terms) than those with a slower *Pace of Life*. Thus, the *Pace of Life* seems to be useful to identify an individual’s stance on the speed-accuracy continuum. In our specific task, placing more weight on speed instead of accuracy paid off: Individuals with a faster *Pace of Life* were ultimately more successful (with regard to their monetary revenue).

## Introduction

When individuals complete tasks, speed often comes at the expense of accuracy: As the literature has shown, individuals tend to make more mistakes the faster they conduct a task [1–3]. Although this speed-accuracy trade-off has been confirmed in the past, there is a lack of evidence on the individual heterogeneity that drives strategic decisions to be either fast or accurate, especially in contrast to external factors like induced time pressure.

We contribute to this research using an approach that is well established in the evolutionary biological literature to analyze a species exposure to time and resulting behavior: the *Pace of Life*. This measure is used to explain differences in the behavior within the species—one animal that can be given here as an example is the snake. Fast-moving snakes are less likely to explore their territory and tend to stay on their habituated paths, whereas slow-moving snakes explore their territory [4]. The *Pace of Life* measure is also used to explain differences in the behavior between species. For example, short-lived animals are more likely to make mistakes than long-lived animals, see e.g. [5]. Recent research shows that it may also drive individual heterogeneity in humans, such as in the decision to speed while driving [6].

In this study, we examined whether the individual *Pace of Life* may serve as a personality trait that explains an individual’s exposure to time and is therefore associated with the speed-accuracy trade-off. Hence, we aimed to understand the following: (1) Do individuals who are characterized by a faster *Pace of Life* accomplish more tasks in total compared to those with a slower *Pace of Life*? (2) Are individuals with a faster *Pace of Life* less accurate (in absolute terms) by making more mistakes compared to individuals with a slower *Pace of Life*? And (3) which strategy proves to be more successful on a certain task—“being fast” or “being accurate”—or something in between?

In a controlled laboratory environment, we quantified the participant’s *Pace of Life* and found that it matched many criteria of a personality trait. Furthermore, we recorded the individual’s performance on an incentivized real-effort task. We found that individuals who had a faster *Pace of Life* solved more tasks in total but also made more mistakes. On this specific task, individuals who placed more weight on speed were monetarily more successful. In particular, we showed that an individual’s *Pace of Life* is a suitable measure to identify heterogeneous types along the speed-accuracy continuum.

## Theoretical background

There are various measures in the existing literature that can help to understand an individual’s behavior. Famous approaches utilize the Big Five personality traits, which contain five characteristics that people can display, e.g. extraversion [7].

Many of these personality or intelligence approaches are self-reported measures [7, 8] and may therefore suffer from many kinds of response bias. Therefore, we used an objective measure by a second person in a clean laboratory setting to avoid certain environmental, subjectively perceived attitudes that could influence the individual’s behavior. Hence, for this study we used the *Pace of Life* approach, which measures walking and working speed with respect to the individual’s objective exposure to time.

### *Pace* *of* *Life*

When focusing on the perception of time, there are different approaches. Time can be seen as subjective as well as objective. Subjective time is the lived or psychological time. This time is processed by the individual human mind [9]. In our study, we shed light on objective time that is measurable, universal and homogeneous, e.g. according to the geographical or clock time that measures time as a physical unit. In this context, we adapted the *Pace of Life* approach given by [10] to measure the individual’s exposure to objective time. [10] used three indicators and built a *Pace of Life* index to identify cultural and economic differences in 31 countries: 1) the average walking speed (which was measured over a distance of 60 feet in downtown locations in each considered city), (2) the working speed (which measured the time it took postal workers to complete a simple request), and (3) the clock accuracy (which measured 15 public clocks in selected downtown banks). In this context, the overall *Pace of Life* index was captured by confirmatory factor analysis using the z-scores for each aggregated measure of individuals in each country.

In contrast to human behavior research, the concept of the *Pace of Life* is well established in biological research [4, 5, 11]. There are only few studies that investigated the relation between individual human behavior and the *Pace of Life*. [12] explored the correlations between personality traits and human metabolism with the aim of reducing measurement noise in the individual *Pace of Life*. Additionally, the study by [6] showed that the *Pace of Life* may also drive individual heterogeneity in humans, such as in the decision to speed while driving. The authors found that individuals with a slower *Pace of Life* were more likely to choose a slow speed in the experiment. These individuals were also more likely to switch to a slow speed, even if they experienced success by driving fast in the preliminary round. However, the empirical evidence on the role of *Pace of Life* for human behavior is rather small so far.

### Speed-accuracy continuum

To maximize economic success in completing tasks which are paid by unit, an individual ideally attempts to work as fast but also as accurate as possible. However, there are situations in which speedy results come at the expense of accuracy and vice versa. In those cases, individuals face a speed-accuracy trade-off [13]. Starting with the work of [1], a trade-off between speed and accuracy was studied in a wide range of settings and experiments. For instance, [2] showed that individuals who performed simple drawing tasks faster were less accurate. In typewriting, an increase in mistakes was associated with more rapid typing [14]. Furthermore, [3] reported that the probability for cricket as well as baseball players to throw a ball accurately decreased with increasing speed. However, the speed-accuracy trade-off is not limited to humans, but can also be found in such animals as house-hunting ants [15], bumblebees [16], guppies [17], mice [18], and monkeys [19].

When it comes to humans, there is statistical evidence that personality traits may sway whether individuals place more value on either speed or accuracy: For example, [20] reported that individuals who had a high level of neuroticism tended to be faster but less accurate in their performance compared to individuals with a low level of neuroticism. Impulsivity was another personality trait that proved to be associated with the speed-accuracy trade-off: individuals with high impulsivity reacted faster but more inaccurately than the less impulsive individuals [21].

However, while established personality traits may help to explain an individual’s behavior along the speed-accuracy continuum, they do not relate directly to an individual’s relation to time. Therefore, we aim to understand whether the individual *Pace of Life* can explain heterogeneity in the speed-accuracy trade-off. Additionally, we want to contribute to this literature by considering whether the individual *Pace of Life* is a potential candidate for a personality trait that refers to the individual relation to (objectively measured) time.

## Methods

We used a controlled laboratory environment to answer our research questions. The study was conducted in the Potsdam Laboratory for Economic Experiments (PLEx) from 12 to 14 October 2020. In total, 11 sessions were carried out and each session consisted of 9 or 10 participants. Ethical approval for this study was obtained by the German Association for Experimental Economic Research. Data are available in the project “Hare and Hedgehog” on OSF: DOI 10.17605/OSF.IO/PSK4H or https://osf.io/psk4h/?view_only=f0900e236c9b4d9db7a70f50badd6128. We used Stata Version 17 for our statistical analysis.

### Participants

We used data provided by [22] to derive a necessary number of about 100 participants to reach a statistical power >0.800. We recruited 110 students from the University of Potsdam, Germany, with the help of the recruitment software ORSEE [23] on a first-come, first-serve basis. Ultimately, 106 students showed up for the experiment. We decided to exclude 9 of the 106 observations, because no reliable measurement of these participants’ *walking time* was possible, e.g. because they walked in pairs (-6 observations), arrived late to the meeting room (-1 observation), or did not follow our route instructions (-1 observations), or there were technical problems in measuring the time (-1 observation). Thus, overall we included 97 observations of German students in our data. The students considered in our data set were between 19 and 36 years old (mean 23.619 and std. dev. 3.898) and 54 (55.670%) were female. Taking 97 observations into our data analysis, we obtained a statistical power between 0.729 and 0.869 assuming an effect size between 5 and 6 (words encrypted in the WEDR task).

### Experimental design

With our experimental design, we aimed to measure the individual speed and accuracy (1), as well as the individual *Pace of Life* of the participants’ (2). To measure the participant’s speed and accuracy (1), we decided to introduce a real-effort task to the participants. Real-effort tasks consist of tasks in which the individual’s performance is monetarily rewarded, and thus incentivized. We chose as a real-effort task the Word Encryption Task with Double Randomization (WEDR task) developed by [22]. Please find the instructions in S1 Appendix. This real-effort task was designed to measure performance by minimizing learning behavior. In the WEDR task, the participants had to encode words. In this context, each word contained three different letters, each associated with a three-digit number. Participants had to find the suitable number from a table displaying all 26 capital letters of the German language and their associated three-digit numbers. Whenever participants encrypted a word correctly, another word was created, new numbers were allocated, and the letters were randomly rearranged; see SI No. 1. Participants could familiarize themselves with the task in a trial stage without financial consequences. For the first 5 words solved correctly, participants received 0.400 EUR each, for words 6–10 they received 0.300 EUR each, for words 11–15 they were paid 0.200 EUR each, and any additional words were remunerated at 0.100 EUR each. The use of decreasing marginal returns is established in economic experiments [24–26], since it reflects an important aspect of production processes in reality. We informed the participants about the payment structure before they conducted the WEDR task. Furthermore, the number of words encoded correctly as well as the individual payoffs were permanently updated and displayed to the participants on their screen during the 15 minutes in which they took part in the WEDR task.

We measured the the number of words encoded incorrectly (errors) and the number of words encoded correctly (achievement). Additionally, we derived a binomial variable that measured whether a participant made incorrect responses at all.

We measured an individual’s *Pace of Life* (2) by capturing different individual aspects related to time. We adapted the approach given by [10], who used walking speed, working speed and clock accuracy to derive a *Pace of Life* index. We recorded the participants’ *walking time* on one section on their way from the meeting room to the PLEx, without any reason to pause. To avoid crowded corridors at the University, the study was conducted during the out-of-session period. Thus, we were able to record unbiased individual *walking time*. The distance measured for *walking time* was 18.900 meters, (about 62 feet) comparable to the distance used in [10].

To quantify the participants’ *working time* for our *Pace of Life* measurement, we let them perform two different tasks of daily life. First, we recorded the time the participants needed to enter a certain number displayed to them using their keyboard—*working time_number_*. This action is usually conducted when logging in to online services like online banking. Secondly, we timed how long the participants needed to fill out a questionnaire—*working time_questionnaire_*, as questionnaires usually have to be completed e.g. for applications, and can thus be considered a common task of daily life too. Thus, both actions considered as measuring *working time* differed from the WEDR task: Individuals had performed them several times previously in their daily lives in a natural environment and the actions were not monetarily incentivized, whereas participation on the WEDR task was new, monetarily incentivized, and associated with a deadline.

These measurements for the *Pace of Life* are objective, since they rely on physical time (clocks). However, in the original *Pace of Life* approach by [10], clock accuracy was also considered for the *Pace of Life* index, but was measured as the objective difference between actual time and public clock time. In this study, we decided not to include clock accuracy as measured by [10], since the participants in our study overwhelmingly used digital watches. Thus, measuring an individual’s clock accuracy would have been meaningless. Nevertheless, we tried to find a suitable replacement for clock accuracy. In doing so, we exploratively recorded the participants’ *inner clock accuracy*. We informed the participants after the WEDR task that they would soon see a blank screen for a certain period of time and had to run their inner clock until the black screen disappeared. During this task, the students were sitting in an empty computer booth, unable to see any clock, and could do nothing other than wait. Afterward, they had to guess how long they had just waited.

### Procedure

**Fig 1:**
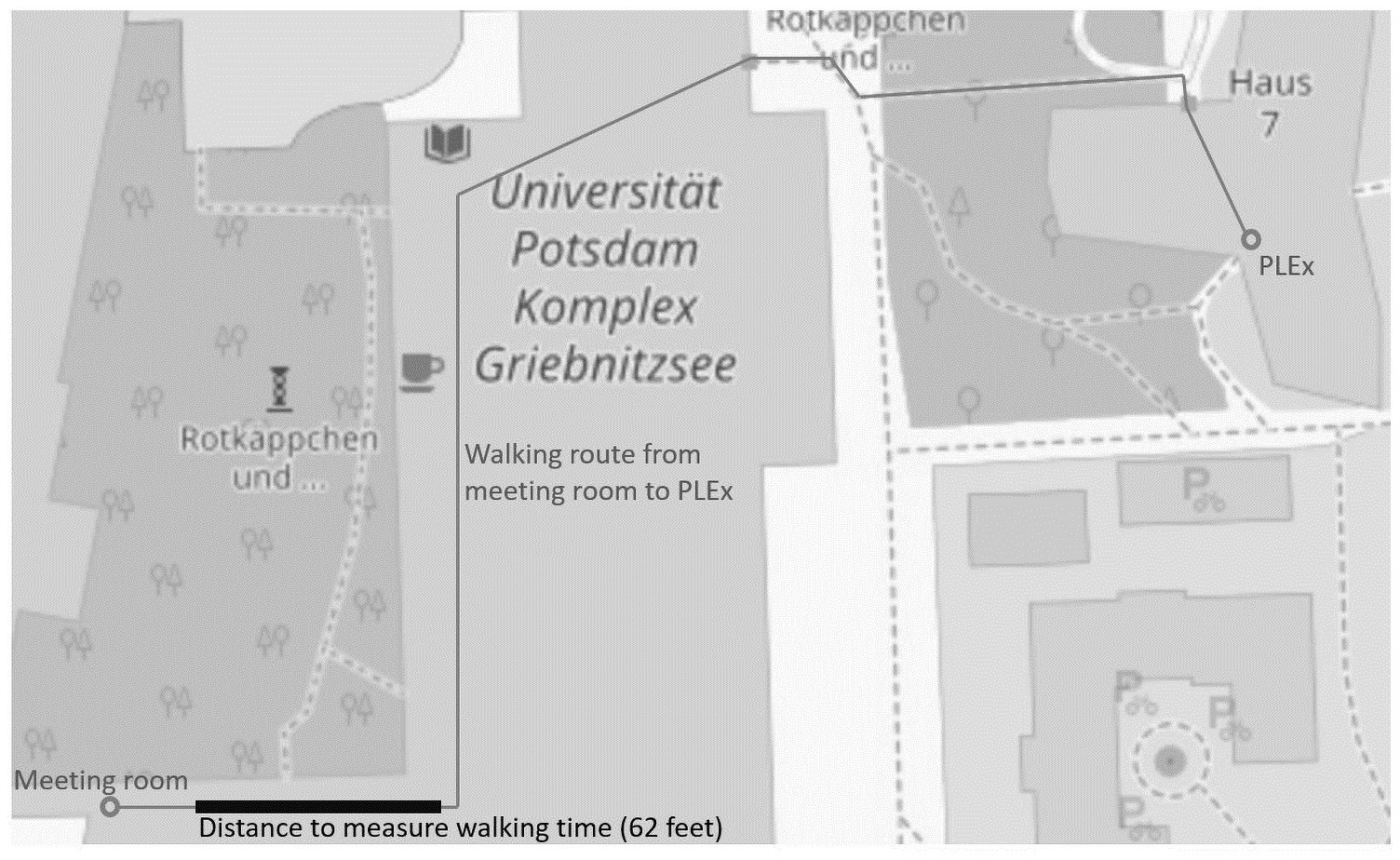
Location of the experiment. Reprinted from OpenStreetMap (https://www.openstreetmap.org/map=18/52.39302/13.12775) under the Creative Commons Attribution License (CC BY 4.0), with permission from © OpenStreetMap Contributers (2020).

Each session started in a meeting room at a certain distance from the PLEx on the campus of the University of Potsdam. In the meeting room, the participants were registered for the experiment and had to give up all their personal belongings, including electronic devices such as smartphones or watches. Afterward, they walked individually and directly to the PLEx. Upon arrival at the PLEx, the participants had to sign an Informed Consent Form. Subsequently, the computer session was initiated. Communication with other participants was not possible during the whole computer session, as students were sitting in empty and separated computer booths. At the end of the experiment—which was computerized with z-Tree [27] -, they received their payment in cash and returned to the meeting room to collect their personal belongings. Please find an illustration of the location in Fig 1.

### *Pace of Life* index

We assumed that the variation in the four variables *walking time*, *working time_number_*, *working time_questionnaire_* and *inner clock accuracy* might reflect the variation in the unobserved latent variable individual *Pace of Life*. Adapting the approach of [28], we used the standardized variables via z-scores and a confirmatory factor analysis to derive an individual *Pace of Life* index. However, we found *inner clock accuracy* to load very poorly in the factor analysis. We ascertained a very low and insignificant correlation between *inner clock accuracy* and the individual *Pace of Life* index (−0.083) if this variable was included (see, S1 Table).

For this reason, and the fact that our measurement of inner clock accuracy was not measured objectively (by physical time), we decided to exclude inner clock accuracy. Instead, we derived the index for the individual *Pace of Life* via confirmatory factor analysis using the three variables *walking time*, *working time_number_*, and *working time_questionnaire_*. Consequently, our *Pace of Life* index was based purely on objective measurements that all correlated significantly with our *Pace of Life* index. Please find factor loadings and the correlation matrix in S1 Table. The overall Kaiser-Meyer-Olkin measure amounted to 0.490, which is slightly below the threshold of 0.6 that should be reached in order to reach a sufficiently measure of sampling adequacy.

**Fig 2:**
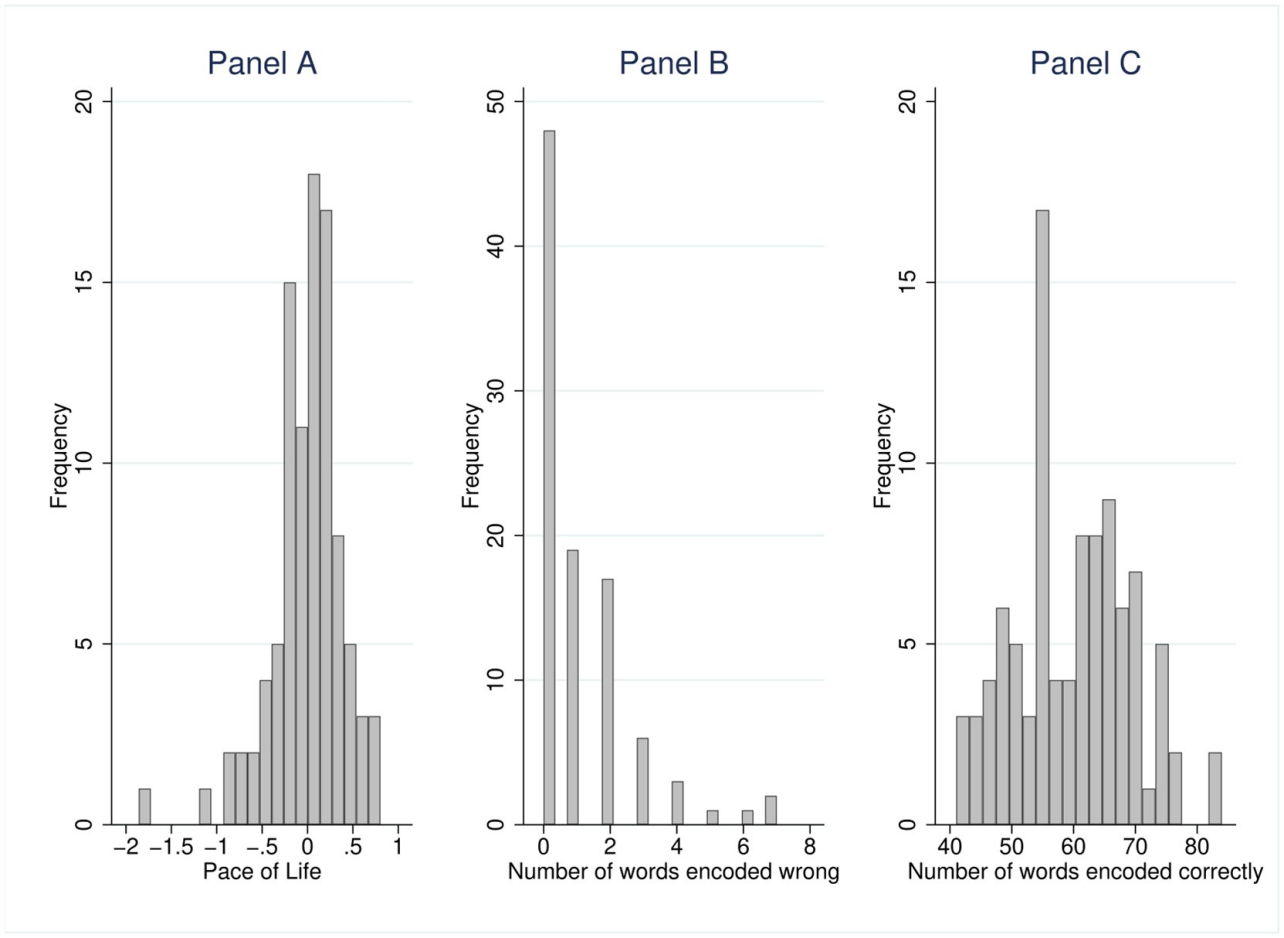
Histogram for the distribution of the variables measuring individual *Pace of Life*, errors and achievement.

Panel A of Fig 2 plots the distribution of the variable individual *Pace of Life*.

The internal consistency, measured by Cronbach’s *α* = 0.304, is comparable to the original *Pace of Life* measurement developed and applied by [10], which is reported as *α* = 0.250. As very early studies rely solely on the walking speed as a measure for *Pace of Life*, like for example [28, 29] argue that especially the modest level of internal consistency points to the importance of capturing multiple facets and improves the measurement of the *Pace of Life* index. Although we reached in our sample a higher *α* than [10], we still see space for improvement in the measurement of the individual *Pace of Life*, as higher level of internal consistency would be desirable.

We contributed to the reliability of our study by analyzing students from diverse fields of specialization with a balanced gender ratio. As we had only one observation per participant, we could not evaluate test-retest stability. However, we tested for within-session reliability of the measured *Pace of Life* using a split-half model. Items of our latent variable were by definition not homogeneous, which made the interpretation as a consistency measure to some extent difficult [30]. However, this type of reliability is especially important throughout the construction phase, as it is not biased by time-dependent changes in the participant’s ability [30]. Assuming that we had two similar forms of the instrument, we calculated the correlation between those two parts. Splitting the construction of the latent variable into the variables *working time_number_* and the *Pace of Life_walking time& working time_questionnaire__*, which is the result of the factor analysis using the variables *working time_number_* and *walking time*, we computed a Spearman-Brown Prophesy Reliability Estimate of 0.419. If we split up *walking time* (or *working time_questionnaire_*), we ended up with a Spearman-Brown Prophesy Reliability Estimate of 0.221 (or 0.224).

Besides the individual *Pace of Life*, there are certainly other socio-demographic characteristics that drive an individual’s success in solving tasks. For instance, age [31–33], income [34, 35], and gender [36–38] might play a role for productivity. We controlled for such effects by asking the participants about their age, gender, and income level (Income category 0 (≤ 500EUR) 26.800%, 1 (501 − 750EUR) 29.900%, 2 (751 − 1000EUR) 26.800%, 3 (1001 − 1250EUR) 8.200% and 4 (> 1250EUR) 8.200% of participants) in a questionnaire.

## Results

Participants solved between 41 and 84 tasks correctly—on average 59.691 (Std.Dev. 9.434). They made between zero and seven mistakes—on average 1.113 (Std.Dev. 1.540). Please find the histograms for the distribution of the variables error rate and achievement in Panels B and C of Fig 2.

### The individual *Pace of Life* and errors

During the 15 minutes of the real-effort task, individuals had to correctly encode as many words as possible to maximize their achievement. In this context, each error, i.e. each word encoded falsely, was associated with a waste of time and no monetary reward.

**Fig 3:**
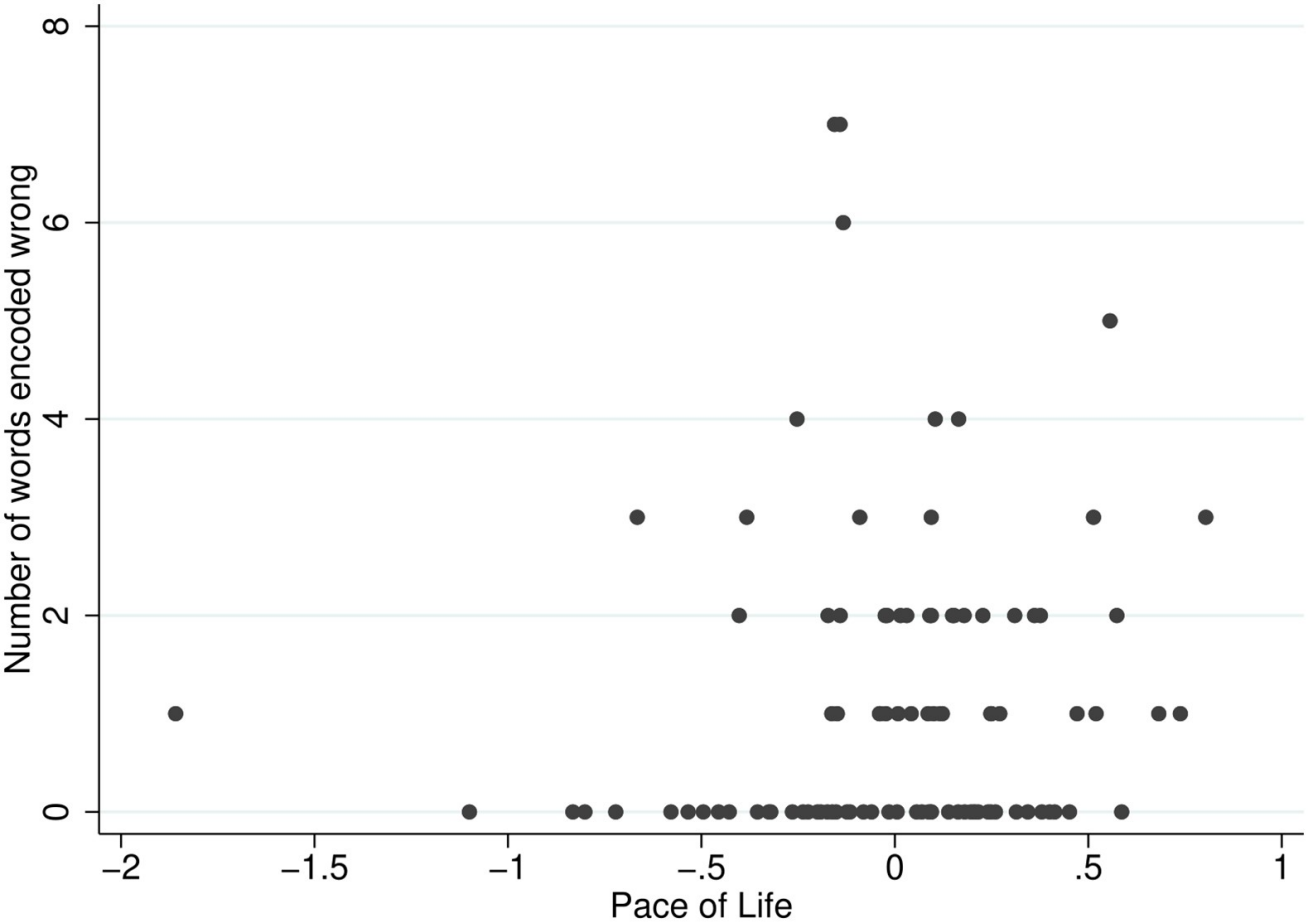
Two-way scatter plot: Number of words encoded incorrectly and *Pace of Life*.

Fig 3 plots the relationship between the *Pace of Life* and the number of incorrect responses.

Next, we analyze the role of the individual *Pace of Life* in increasing the errors—which are the number of incorrect responses—by running a multiple, ordinary least squares regression.

Therefore, we estimate the following basic equation:

$$\begin{array}{c} Errors_{i}=\alpha_{i}+\beta_{i}^{Pace\;of\;Life}Pace\;of\;Life_{i}+\beta x_{i}+\epsilon_{i} \end{array}$$ (1)

for *i* = 1, …, *n* participants, where *Errors*_*i*_ is measured in number of mistakes made on the real-effort task, i.e. words encoded incorrectly, and **x**_**i**_ is the vector of control variables, i.e. age, gender, and income categories, as well as session and day dummies. The main coefficient of interest $\beta_{i}^{Pace\;of\;Life}$ measures the relationship between a participant’s *Pace of Life* and the errors. We apply a step-up approach and present in Table 1 the estimation results for Eq 1.

**Table 1:** Regression results for errors.

	Number of incorrect responses					Making incorrect responses at all				
	(1)	(2)	(3)	(2a)	(3a)	(4)	(5)	(6)	(5a)	(6a)
*Pace of Life*		0.780*		0.925**			0.949**		1.533***	
		(0.400)		(0.448)			(0.467)		(0.470)	
*Fast Pace of Life*			0.096		0.085			0.337		0.355
			(0.355)		(0.354)			(0.304)		(0.303)
Female	-0.476	-0.477	-0.478	-0.490	-0.470	-0.021	-0.001	-0.020	-0.051	-0.036
	(0.349)	(0.349)	(0.350)	(0.349)	(0.352)	(0.290)	(0.298)	(0.292)	(0.303)	(0.291)
Age	-0.044	-0.044	-0.045	-0.044	-0.045	-0.040	-0.042	-0.046	-0.045	-0.047
	(0.040)	(0.039)	(0.039)	(0.039)	(0.039)	(0.038)	(0.038)	(0.038)	(0.039)	(0.038)
Constant	1.780	1.572	1.766	1.562	1.752	1.138	0.926	1.097	0.947	1.129
	(1.164)	(1.150)	(1.180)	(1.149)	(1.182)	(1.190)	(1.213)	(1.217)	(1.248)	(1.215)
*N*	97	97	97	96	96	97	97	97	96	96
*R* ^2^	0.181	0.211	0.182	0.214	0.183					
*PseudoR* ^2^						0.099	0.143	0.108	0.172	0.105

Regressions additionally include session-day dummies, and income category as a control variable, Standard errors in parentheses,

* *p* < 0.100,

** *p* < 0.050,

*** *p* < 0.010.

In model (1), we estimate Eq 1 without the variable of interest. In model (2), we add the variable *Pace of Life* and find a positive and significant relationship between the individual *Pace of Life* and the number of mistakes in model (2). An increase in the *Pace of Life* of one unit increases the number of mistakes by about 0.780. To allow for a more convenient interpretation of our results, we again use the binary variable *Fast Pace of Life* in model (3).

About 50% of our participants made no mistakes at all. To account for this censoring in the data structure, we additionally apply a probit model. Deriving marginal effects at the means of our participants, we estimate that if the individual’s *Pace of Life* increases by one unit, the probability of making mistakes at all increases significantly by about 32.131%, see model (4). In model (5), we again replace the cardinal variable *Pace of Life* with the binary one *Fast Pace of Life*. Estimation results are of the expected sign and size, but not significant. This result may be driven by the fact that we reduced the degree of information by implementing the median split.

We additionally show in models (2a & 3a) as well as (5a & 6a) that the results of the relationship between the individual *Pace of Life* and errors are robust to the removal of one extreme outlier.

### The individual *Pace of Life* and achievement

**Fig 4:**
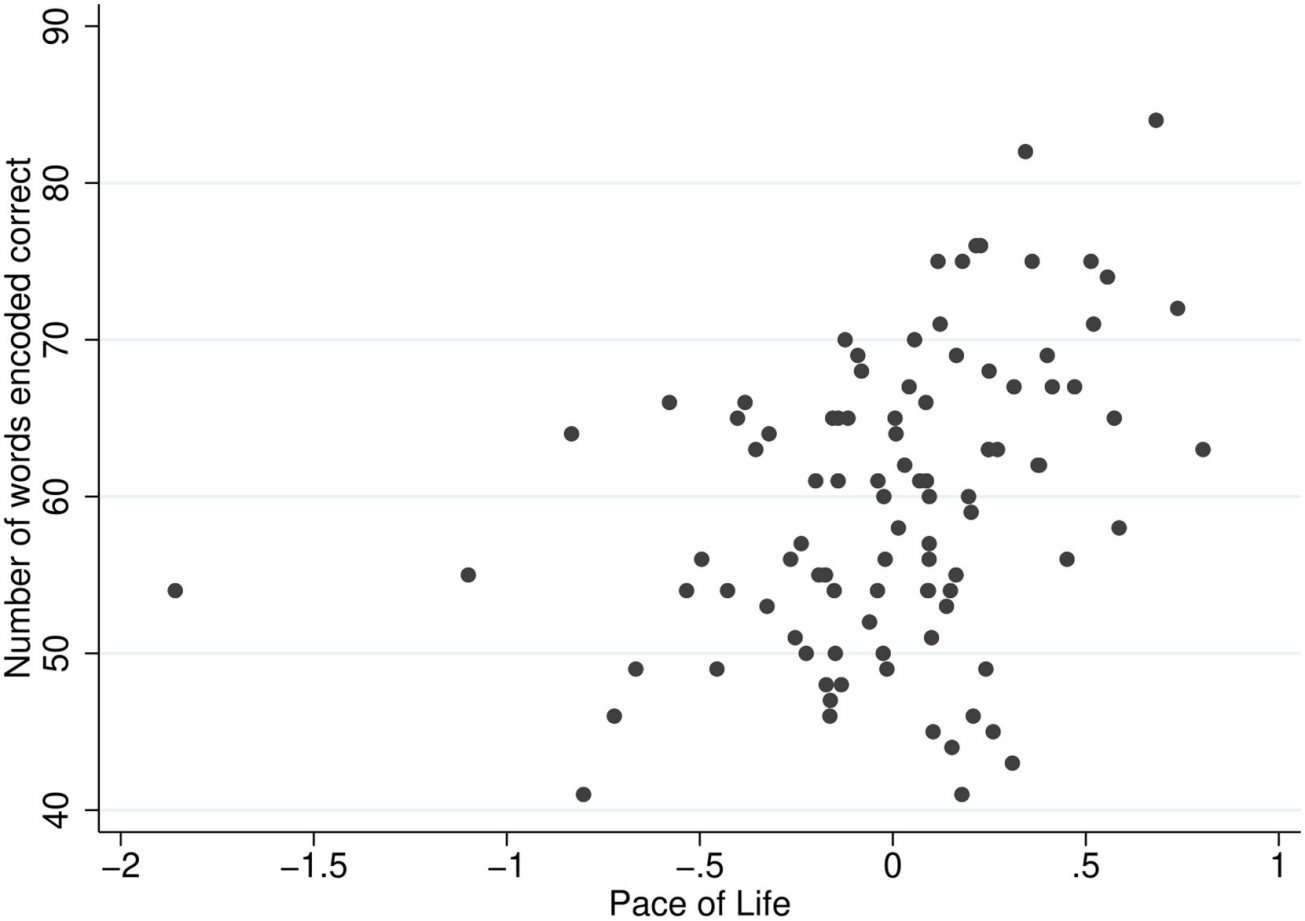
Two-way scatter plot: Words encoded correctly and *Pace of Life*.

We measure an individual’s achievement by the number of words encoded correctly in 15 minutes of working time. Fig 4 plots the relationship between the individual *Pace of Life* and the achievement reached on the real-effort task.

We again run several ordinary least square regressions to analyze the relationship between the individual *Pace of Life* and achievement on the real-effort task of the experiment. Therefore, we estimate the following basic equation:

$$\begin{array}{c} Achievement_{i}=\alpha_{i}+\beta_{i}^{Pace\;of\;Life}Pace\;of\;Life_{i}+\beta x_{i}+\epsilon_{i} \end{array}$$ (2)

for *i* = 1, …, *n* participants, where *Achievement*_*i*_ is measured in the number of words encoded correctly on the real-effort task or the payoff received for the real-effort task respectively; **x**_**i**_ is the vector of control variables, i.e. age, gender, and income categories, as well as session and day dummies. The main coefficient of interest $\beta_{i}^{Pace\;of\;Life}$ measures the relationship between a participant’s *Pace of Life* and achievement. We apply a step-up approach and present in Table 2 the estimation results for Eq 2.

**Table 2:** Regression results for achievement.

	Words encoded correctly					Payoff		
	(1)	(2)	(3)	(2a)	(3a)	(4)	(5)	(6)
*Pace of Life*		10.171***		14.253***			101.713***	
		(3.333)		(2.230)			(33.330)	
*Fast Pace of Life*			6.434***		6.559***			64.340***
			(1.953)		(1.961)			(19.530)
Female	1.946	1.939	1.848	1.563	1.764	19.463	19.387	18.483
	(2.079)	(1.821)	(1.900)	(1.764)	(1.915)	(20.793)	(18.210)	(19.003)
Age	0.315	0.313	0.197	0.306	0.193	3.146	3.130	1.967
	(0.340)	(0.290)	(0.303)	(0.278)	(0.304)	(3.398)	(2.896)	(3.029)
Constant	56.066***	53.353***	55.107***	53.053***	55.263***	860.655***	833.532***	851.075***
	(11.319)	(9.754)	(10.279)	(9.238)	(10.294)	(113.187)	(97.539)	(102.792)
*N*	97	97	97	96	96	97	97	97
*R* ^2^	0.197	0.333	0.288	0.397	0.289	0.197	0.333	0.288

Regressions include session-day dummies and income category as a control variable. Standard errors in parentheses,

* *p* < 0.100,

** *p* < 0.050,

*** *p* < 0.010

In model (1), we estimate Eq 2 without the variable of interest. In model (2), we find a large, positive, and highly significant relationship between the *Pace of Life* and the number of words encoded correctly. An increase in the *Pace of Life* of 1 unit increases the number of words encoded correctly by about 10.171.

For a more convenient interpretation of these results, we again replaced the continuous variable *Pace of Life* with the binary one *Fast Pace of Life*. The result is presented in model (3). Participants with a *Fast Pace of Life* correctly encode about 6.400 words more than participants with a *Slow Pace of Life*. This estimation result is statistically significant.

Additionally, we show in Table 2, models (2a) and (3a), that our results are robust to the exclusion of an extreme outlier with a very low individual *Pace of Life*. Without this extreme observation, results are even slightly stronger.

As the outcome variable achievement is also the one that is used to compute the payoff the participants received from the laboratory study, we additionally report regression results with the dependent variable payoff, which is a linear transformation of the number of words encoded correctly.

Additionally, we ran each regression again using the components of the latent variable *Pace of Life* instead of the *Pace of Life* itself as an explaining variable. All estimates were of the expected direction. Emerging from significance levels, *working time_questionnaire_* seemed to have the most consistent relation to all three dependent variables. Please find the results in the S2 Table.

## Discussion and conclusion

The literature has shown that individuals make different decisions if they encounter a speed-accuracy trade-off in solving tasks. In general, some individuals place more value on being *fast*, taking mistakes into account, while others focus more on being *accurate*, taking slowness into account. There are also individuals who balance speed and accuracy when accomplishing tasks. Crucial for the individuals’ strategy and their position on the speed-accuracy continuum are e.g. incentives. Against this background, we studied whether the *Pace of Life* approach introduced by [10] fulfills the criteria of a personality trait and might explain differences between the two strategies—being fast or being accurate.

While this concept has rarely been studied in humans, it is used extensively in the biological literature. In this literature, the *Pace of Life* strongly relates to the life-history continuum, presuming that between- and within-species variation in physical attributes like metabolism determines variation in behavior [5]. In this study, we applied a novel measurement of the *Pace of Life* and conducted a certain task which is characterized by a speed-accuracy trade-off to test whether the *Pace of Life* may predict the individual’s stance in the speed-accuracy continuum in our setting.

In a controlled laboratory experiment, we examined the participants’ *Pace of Life* by recording walking time and several aspects of working time. Furthermore, we measured the accuracy of their answers and their resulting achievement on a real-effort task.

The *Pace of Life* measurement considered in this study fulfills many criteria of a personality trait. Personality traits refer to characteristics that individuals have in common, whereby individuals differ with regard to the extent of these characteristics. The latter makes it possible to compare individuals [39]. In our study, we observed the behavior of individuals in three situations that are common in the daily life of all individuals to derive the individual *Pace of Life*: walking a certain section, completing an action that was comparable to logging in to online services, and answering a questionnaire. In this context, we ascertained that the individual *Pace of Life* differed across participants.

Furthermore, it is assumed that personality traits are relatively stable over time, show internal consistency, and influence behavior in certain situations [40]. Stability over time could be neither confirmed nor rejected with our study design. However, our results showed that the three measurements that formed our *Pace of Life* index all correlated significantly with the *Pace of Life* index. Furthermore, the individual *Pace of Life* affected the behavior on the real-effort task that we conducted and proved to have a significant impact regarding the individual’s stance on the speed-accuracy continuum.

By applying a typical real-effort task that is repeatedly used in economics, we found that individuals with a slower *Pace of Life* solved fewer tasks in total but were more accurate compared to individuals with a faster *Pace of Life*. We conclude that an individual’s *Pace of Life* may serve as an appropriate approach to identify the individual’s type in terms of position along the speed-accuracy continuum at this specific task.

Here, making mistakes was merely associated with a waste of time but no financial disincentives. Thus, by placing more weight on speed instead of accuracy, individuals with a faster *Pace of Life* were ultimately more successful from a monetary point of view. On tasks with pay-off functions that penalize mistakes more heavily, individuals with a slow *Pace of Life* may be superior. However, the means by which the relationship between heterogeneity in the *Pace of Life* and achievement on real-effort tasks depends on different pay-off functions remains a subject for future research.

Although we constructed the *Pace of Life* index carefully and with consideration of the concept that is established on the macro-scale, (see e.g. [10, 28, 41, 42]), there are some possible limitations. The first variable *walking time* was measured by research assistants using electronic time clocks. While we made sure that the same person always measured the *walking time* of individuals walking the same distance alone, using the same clock, there may have been result small measurement errors. Using light barriers instead could be a possible improvement of this method. If a reliable measurement of *walking time* was not guaranteed, which was for the case when participants walked in pairs, for example, or did not follow our route instructions, or arrived late to the meeting room, we excluded these observations.

In measuring *working time*, we may find some limitations if participants had different experiences performing the daily life tasks we chose. However, as we had a very homogeneous set of participants with respect to age, education, and origin, we reduced the possible variability in these variables to a minimum.

Furthermore, given the fact that a share of our participants did not make any incorrect response at all, it may be the case that some participants just worked faster, irrespective of their level of accuracy or that their cognitive abilities differed. Future research should address these aspects by controlling for the participant’s intelligence.

## Supporting information

S1 Fig Example for the trial stage of the word encryption task. (TIF)

S1 Appendix Instructions for participants of the experiment. (PDF)

S1 Table Correlation matrix: Individual *Pace of Life* and components. (ZIP)

S2 Table Regression results for components of *Pace of Life*. (ZIP)
