# Supplementary figures and images for: The Hare and the Hedgehog: Empirical evidence on the relationship between the individual Pace of Life and the speed-accuracy continuum
Source: PLoS One. 2021 Aug 20;16(8):e0256490. doi: 10.1371/journal.pone.0256490 (PMC8378698; doi:10.1371/journal.pone.0256490)

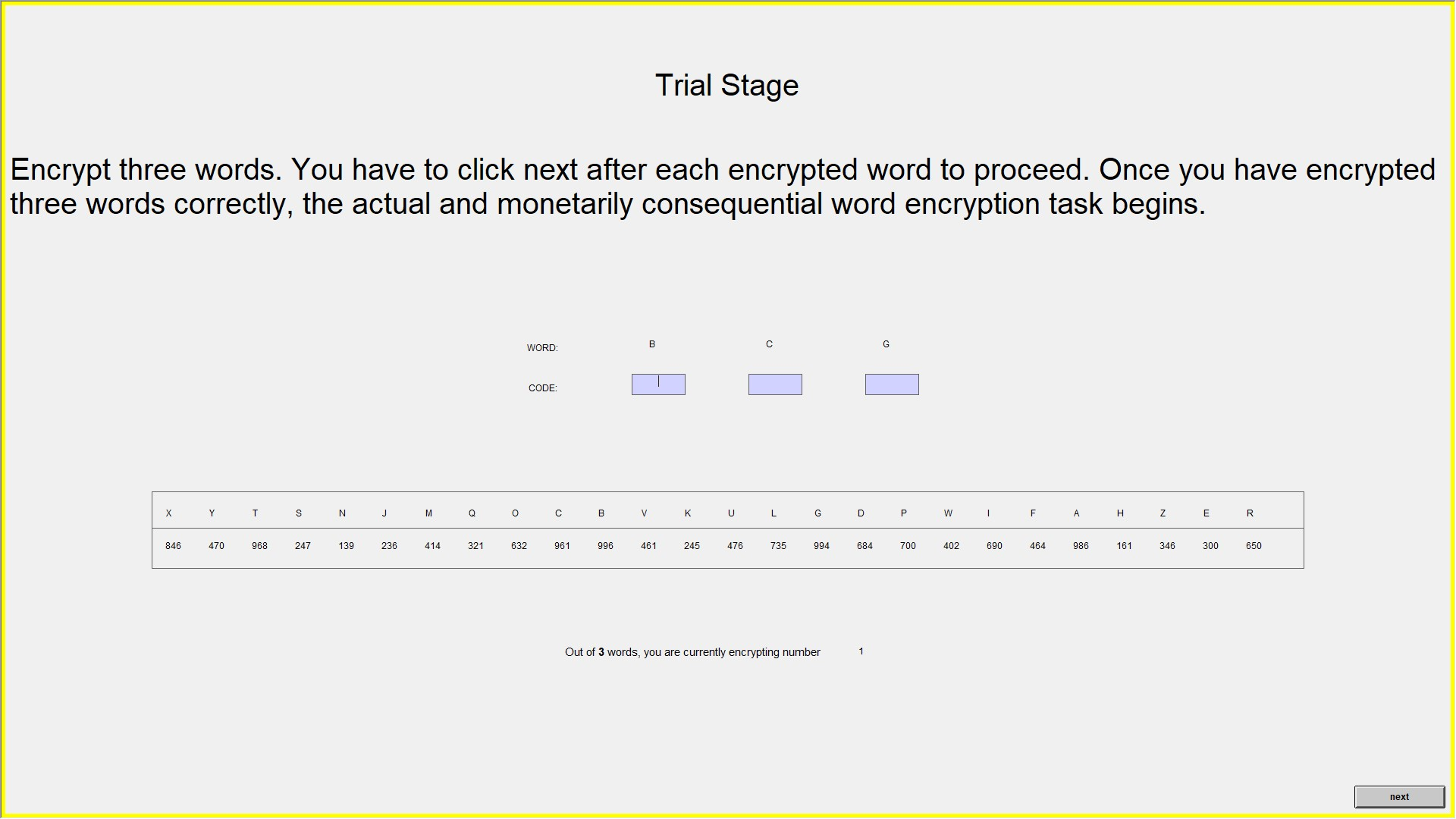

Supplement: S1 Fig — (TIF) [file pone.0256490.s001.tif]
