# Supplementary material for: The Hare and the Hedgehog: Empirical evidence on the relationship between the individual Pace of Life and the speed-accuracy continuum
Source: PLoS One. 2021 Aug 20;16(8):e0256490. doi: 10.1371/journal.pone.0256490 (PMC8378698; doi:10.1371/journal.pone.0256490)
Supplement: S1 Appendix — (PDF) [file pone.0256490.s002.pdf]

SI 1 Appendix

The section provides the English translation of the original German instructions which the participants at PLEx received before their experiment started on the screen.

Instruction

Welcome to the experiment.

We ask you to stay seated and stop communicating with the other participants as of now. Please turn off your mobile and electrical devices. It is of the utmost importance to adhere to the rules. Otherwise, we reserve the right to exclude you from the experiment and, with that, from the payment.

Every participant has received identical instructions. Please read these instructions very carefully. Raise your hand in case you have any questions. We will answer questions personally.

By participating in this experiment, you can earn money. You have already collected 5 EUR by arriving on time. This experiment is about a word encryption task. It takes exactly 15 minutes. In the course of this exercise, you will be shown words to encrypt.

Every word consists of three letters. Each letter has a matching numerical code, which you have to find in the chart below. You need to enter this code in the empty space underneath the letters. If you execute this task correctly for all three letters, you have successfully encrypted a word, earning an additional amount of money. After that, you will see a new chart and a new word to decode.

For each encrypted word from 1 to 5, you receive 40 cents. For the encrypted words 6 to 10, you receive 30 cents. For the encrypted words 11 to 15, you receive 20 cents, and from the 16th word on, you receive 10 cents per word encrypted correctly.

Following the word encryption task, you will take part in a time estimation task. The time will be counted down invisibly from a certain moment. Afterward, we will ask you to estimate how much time has passed. You can also earn money with your estimation: If your estimation is accurate, your payment will be raised by 2 EUR. For every second your estimation differs from the actual time, you lose 10 cents. Hence, you will not receive an additional payment in this task if you deviate more than 20 seconds from the actual time.

Overall, your profit consists of the 5 EUR for showing up on time, the amount earned on in the word decoding task, and your bonus for accuracy on the time estimation task.

At the end of the experiment, you can decide whether and how much of your earned money you would like to donate to a tree-planting project. After that, we will ask you to complete a questionnaire. Finally, we will direct you individually to the payment counter.
