# Supplementary material for: The Hare and the Hedgehog: Empirical evidence on the relationship between the individual Pace of Life and the speed-accuracy continuum
Source: PLoS One. 2021 Aug 20;16(8):e0256490. doi: 10.1371/journal.pone.0256490 (PMC8378698; doi:10.1371/journal.pone.0256490)
Supplement: S1 Table — (ZIP) [file pone.0256490.s003.zip › S1_Table.pdf]

S1 Table.

Table 1. Correlation matrix: The individual *Pace of Life* and its components

	Factor loadings	*Pace of Life*	*Fast Pace of Life*	*walking time*	*working* $time_{number}$	*working* $time_{quest.}$
Pace of Life		1				
Fast Pace of Life		0.718^{***}	1			
walking time	0.274	-0.308^{**}	-0.287^{**}	1		
working $time_{number}$	0.412	-0.780^{***}	-0.448^{***}	0.187	1	
working $time_{quest.}$	0.279	-0.735^{***}	-0.622^{***}	0.005	0.189	1

Standard errors in parentheses * $p < 0.05$, ** $p < 0.01$, *** $p < 0.001$
