# Supplementary material for: The Hare and the Hedgehog: Empirical evidence on the relationship between the individual Pace of Life and the speed-accuracy continuum
Source: PLoS One. 2021 Aug 20;16(8):e0256490. doi: 10.1371/journal.pone.0256490 (PMC8378698; doi:10.1371/journal.pone.0256490)
Supplement: S2 Table — (ZIP) [file pone.0256490.s004.zip › S2_Table.pdf]

S2 Table.

Table S2. Regression results for components of *Pace of Life*

	Number of Mistakes			Making Mistakes at all			Achievement		
walking time	-0.222			-0.141			-1.685*		
	(0.179)			(0.123)			(0.929)		
$working\ time_{number}$		-0.016			-0.009			-0.200	
		(0.012)			(0.012)			(0.134)	
$working\ time_{quest.}$			-0.003			-0.006***			-0.043***
			(0.003)			(0.002)			(0.012)
Female	-0.318	-0.439	-0.564	0.073	0.005	-0.188	3.148	2.431	0.612
	(0.395)	(0.352)	(0.372)	(0.304)	(0.290)	(0.318)	(1.902)	(1.966)	(1.966)
Age	-0.048	-0.045	-0.041	-0.041	-0.041	-0.039	0.285	0.300	0.348
	(0.039)	(0.039)	(0.040)	(0.038)	(0.037)	(0.039)	(0.324)	(0.315)	(0.314)
Constant	4.171*	2.148*	2.619**	2.623	1.359	3.066**	74.229***	60.815***	68.893***
	(2.356)	(1.174)	(1.308)	(1.847)	(1.212)	(1.501)	(14.740)	(10.938)	(11.027)
N	97	97	97	97	97	97	97	97	97
R^2	0.203	0.194	0.196				0.231	0.254	0.290
$Pseudo R^2$				0.107	0.103	0.166			

All regressions additionally include session-day dummies and income category as a control variable.
Standard errors in parentheses, $^{*}p < 0.100, ^{**}p < 0.050, ^{***}p < 0.010$
